# The effectiveness of an actuator-driven pulsed water jet for the removal of artificial dental calculus: a preliminary study

**DOI:** 10.1186/s12903-020-01190-8

**Published:** 2020-07-13

**Authors:** Yuka Sato, Masahiro Iikubo, Takashi Nishioka, Nobuhiro Yoda, Tetsuya Kusunoki, Atsuhiro Nakagawa, Keiichi Sasaki, Teiji Tominaga

**Affiliations:** 1 Division of Dental Informatics and Radiology, Tohoku University Graduate School of Dentistry, 4-1 Seiryo-machi, Aoba-ku, Sendai, Miyagi 980-8575 Japan; 2 Division of Advanced Prosthetic Dentistry, Tohoku University Graduate School of Dentistry, Sendai, Miyagi Japan; 3 Department of Neurosurgery, Tohoku University Graduate School of Medicine, Sendai, Miyagi Japan

**Keywords:** Actuator-driven pulsed water jet, Dental calculus, Tooth surface

## Abstract

**Background:**

While hand and ultrasonic scalers are the primary tools used for the removal of dental calculus in periodontal treatment, many studies have shown that they also damage the enamel surface. We have developed a novel actuator-driven pulsed water jet (ADPJ) system, which has the ability to selectively remove materials depending on their stiffness. Considering the different material properties between teeth and dental calculus, it might be possible to develop the ADPJ to remove dental calculus without damage to the tooth’s enamel surface using a suitable jet pressure. Therefore, the aim of this study was to assess the effectiveness of the ADPJ in removing dental calculus, and the surface features of the teeth after its use.

**Methods:**

A total of 93 artificial teeth coated with artificial dental calculus were examined in this study. The weights of 90 teeth were measured before and after the use of ADPJ, which had an applied voltage setting of 150, 200, or 240 V. The three remaining teeth were instrumented with a conventional hand scaler, ultrasonic scaler, or ADPJ (set at 240 V). Damage to the artificial tooth surfaces was evaluated using 5% Evans blue dye under an optical microscope. Furthermore, apatite pellets, which are utilized as experimental substitutes for natural teeth, were assessed after the use of ADPJ and both conventional scalers.

**Results:**

The ADPJ significantly reduced the amount of artificial calculus, and the removal rate was dependent on the applied voltage. No damage was observed on the surface of the artificial tooth and apatite pellet following the use of ADPJ, in contrast to the conventional scalers.

**Conclusions:**

The results of this study demonstrate the in vitro effectiveness of ADPJ in the removal of dental calculus, without causing damage to tooth surfaces.

## Background

Dental calculus is formed supra- and sub-gingivally, and contributes to the irritation and inflammation of the gingiva; this in turn leads to gingivitis and periodontitis. Removal of dental calculus is the mainstay of prevention and treatment of periodontal disease [1], and this is most commonly accomplished with the use of ultrasonic or hand scalers [2]. Especially, ultrasonic scalers are often used, owing to their simplicity, efficiency, and the wide range of designs available for accessing different anatomical areas. However, many studies have reported that both ultrasonic and hand scalers cause damage to the enamel surface, especially the ultrasonic scaler makes deep damage on the crack line of the enamel surface and crater formation with remarkable disintegration of the demineralized enamel surface [3, 4]. Some studies have reported the use of an erbium-doped yttrium aluminum garnet laser (Er: YAG laser) with high pulse repetition rates (focused on a small area) to selectively remove dental calculus with high precision [5–7], with limited damage to the sound tissues [6, 7]. Nevertheless, the guideline has stated that the use of lasers to remove dental calculus is contraindicated due to the risk of thermal damage and ablation of enamel [6]. Therefore, the development of a device that can safely remove dental calculus without damaging the enamel surface is required.

We have previously developed an actuator-driven pulsed water jet (ADPJ) system which removes lesions, such as tumors, with minimal damage to the surrounding healthy tissues [8]. The ADPJ is an emerging technology with a remarkably reduced water consumption and has the ability to selectively remove tissues based on their material stiffness [8–10]. Considering the different material properties of enamel and dental calculus, it might be possible to develop the ADPJ to use appropriate water pressure to remove dental calculus without damaging the underlying enamel surface. Since the water pressure of the ADPJ was consistently and positively correlated with the applied voltage [9, 10], we subsequently modified our original ADPJ and developed a device with a higher applied voltage, with the aim of removing harder materials such as dental calculus. The objective of this study was to assess the effectiveness of this modified ADPJ system at different applied voltage settings, in removing dental calculus using an artificial model. Furthermore, we assessed the ability of the ADPJ to limit damage to enamel surfaces, in comparison to conventional scalers.

## Methods

### ADPJ system

**Fig. 1:**
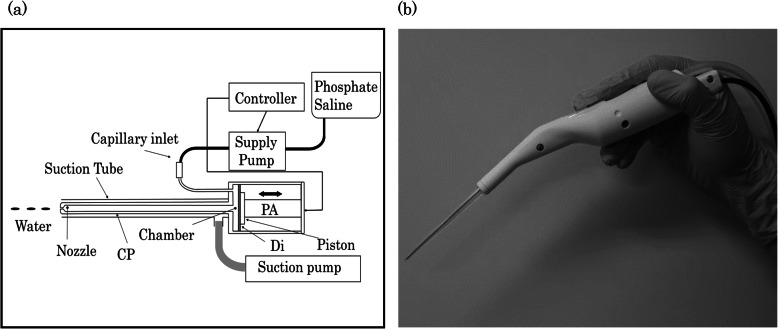
Schematic diagram (**a**) and photograph (**b**) of the piezo ADPJ system. The ADPJ is generated by the movement of piston driven, the water is released through the chamber continuously. The three-dimensional water flow ejected from the nozzle can be adjusted with a manual switch, and the water volume and speed are easy to control. Dissected tissue and splashed and excess liquid are aspirated through the removable silicone suction tube connected to a conventional aspirating system in the operating theater. ADPJ: actuator-driven pulsed water jet, CP: stainless steel connecting pipe, Di: stainless steel diaphragm, PA: piezo electric actuator

We have previously described the mechanism of the ADPJ system [8–10]. In brief, this system is composed of a pump chamber driven by a piezo-actuator, a stainless steel tube, and a nozzle with an internal diameter of 0.15 mm, as shown in Fig. 1. Pure water is continuously fed into a chamber that supplies the water pump through a capillary inlet (inner diameter, 0.3 mm) at a flow rate of 12.4 mL/min. The piston driven by the piezo-actuator compression occurs from 0 to 125 μs after a sinusoidal wave pattern with a phase angle of −π/2 to π/2. Piston recovery occurs gradually from 125 to 5000 μs after a low-frequency sinusoidal wave pattern. The resultant piston is moved at a frequency of 200 Hz. The piston pushes the diaphragm forward to compress the chamber and generates pressure. The elevated pressure in the chamber propagates to the nozzle through the connecting pipe. Water ejected from the nozzle can be removed through the suction tube.

In our previous ADPJ system, the peak-to-peak value of the applied voltage that drove the piston was set at 5 to 100 V [8–10]. In this experiment, the maximum voltage was increased to 240 V, with the aim of removing dental calculus, which is stiffer than the previously tested materials.

### Mechanical properties of the water jet

The mechanical properties of the ADPJ system were evaluated by measuring the peak pressure with a cavity-mounted pressure sensor (Quartz High-Pressure Sensor, Model 601A; Kistler Instrumente AG, Winterthur, Switzerland). The length of the sensing hole was 0.5 mm and the diameter was 0.15 mm, which was equivalent to that of the nozzle diameter of our ADPJ system. The sensing hole was tapered and connected to the cavity (1.5 mm length), which had the same diameter as the pressure sensor. The nozzle of the ADPJ system was placed 0.5 mm from the sensing hole and the pressure was measured accordingly. The tip of the cavity-mounted pressure sensor unit and the ADPJ system was placed in a water reservoir from which air was extracted to avoid the formation of bubbles. The peak pressure of the ADPJ was measured three times for each applied voltage setting (150 V, 200 V and 240 V).

### Preparation of the dental calculus samples

**Fig. 2:**
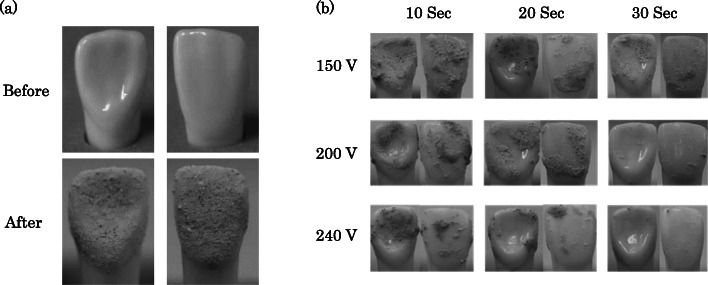
Coating of the artificial teeth with artificial calculus, and removal using the ADPJ. **a** Before and after coating of the artificial teeth with artificial calculus. **b** The residual artificial calculus after treatment with the ADPJ under each voltage setting and treatment duration combination. ADPJ: actuator-driven pulsed water jet

Before the experiment, the weight of each artificial tooth (initial weight) was measured using a standard-level precision balance (FX-300i, Kensei Kogyo Co., Ltd., Ibaraki, Japan). As shown in Fig. 2a, the artificial dental calculus (Dental Calculus Set; Nisshin Dental Products Inc. Tokyo, Japan) was coated over the entire surface of each artificial right upper primary incisor crown (*n* = 93) (Invictus, A-PRO3A; Nisshin Dental Products Inc., Tokyo, Japan) with a uniform thickness. The teeth were stored for over 24 h in a controlled environment at 24 °C before being subjected to the ADPJ.

### Evaluation of the effectiveness of artificial dental calculus removal

After the weights of the samples (weight before ADPJ) were measured, the artificial dental calculus was removed with the ADPJ system set at a voltage of either 150 V, 200 V, or 240 V, and for a duration of either 10 s, 20 s, or 30 s. Ten trials, in which the nozzle of ADPJ was placed perpendicular to the artificial dental calculus, at a distance of approximately 1.5 mm, and ran in parallel to the artificial tooth surface, were performed for each combination of treatment duration and applied voltage setting, for a total of 90 trials. The weight of each artificial tooth (weight after ADPJ) was measured again after a period of at least 24 h in a controlled environment at 24 °C. The removal rate (%) of the artificial dental calculus was analyzed based on the following formula: (weight before ADPJ – weight after ADPJ) × 100 / (weight before ADPJ – initial weight).

### Evaluation of the surface of the artificial teeth and apatite pellets

The artificial dental calculus of three samples was removed by either a conventional hand scaler (No.23326 G6, YDM Co. Tokyo, Japan), ultrasonic scaler (0E5, NSK Ltd., Tokyo, Japan), or ADPJ (set at 240 V) for 1 min. The conventional hand scaler and ultrasonic scalers were used by a dentist with more than 20 years of experience. The artificial teeth were immersed in 5% Evans blue dye solution for 10 s, and rinsed with water for another 10 s. The buccal surfaces of the artificial teeth were evaluated under an optical microscope (TG500PC, Shodensha, Inc., Osaka, Japan).

Apatite pellets (APP-100; 10 × 10 × 2 mm, Hoya Technosurgical Co., Tokyo, Japan), which are utilized as experimental substitutes for natural teeth in studies investigating the abrasiveness of toothbrushes and tooth paste, were polished in one direction to evaluate the surface properties; they were then attached to a stage moved in a direction perpendicular to that in which they were polished (EZSM 3E020-K, Oriental Motor Co., LTD, Tokyo, Japan) [11]. The nozzle of the ADPJ was placed 1.5 mm from the apatite pellet, and the applied voltage was set to 240 V. The apatite pellet was subsequently moved 10 mm at a speed of 0.5 mm/s. Ultrasonic and hand scalers were pressed against the apatite pellet with a force of 0.4 N, 0.6 N or 0.8 N; this was performed after both scalers were placed on an electronic balance and the pressure was adjusted [12]. The scratch on the apatite pellet surface was evaluated by laser microscopy (VK-9500, Keyence Co., Inc., Osaka, Japan).

### Statistical analysis

All values are presented as means ± standard error. Statistical analyses were performed using JMP 13 Pro statistical software (SAS Institute Inc., Cary, NC, USA). Comparisons of the nine groups were performed using a one-way analysis of variance test and Tukey-Kramer HSD test. The level of statistical significance was set at *p* < 0.05.

## Results

### The effectiveness of artificial calculus removal with ADPJ under each condition

**Fig. 3:**
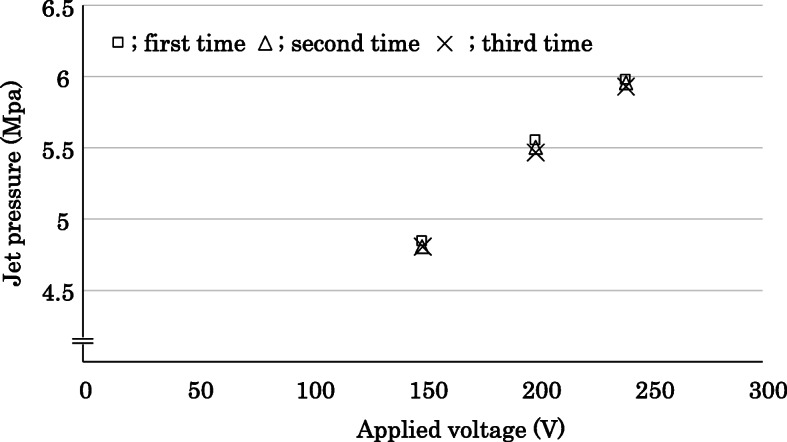
Relationship between the ADPJ voltage setting and peak water jet pressure. X-axis is the applied voltage (V). Y-axis is the jet pressure (Mpa). ADPJ: actuator-driven pulsed water jet

As shown in Fig. 3, the peak pressure of the pulsed water jet was positively correlated with the applied voltage (R^2^ = 0.9943, *p* < 0.001).

The mean adherent weight of the artificial dental calculus (weight before ADPJ – initial weight) was 22.2 ± 6.8 mg and there was no significant intergroup difference. Figure 2b depicts typical samples of artificial teeth after ADPJ treatment under the three applied voltage settings (150 V, 200 V, and 240 V) and three treatment durations (10 s, 20 s, and 30 s). The artificial dental calculus was partially removed from the surface of the artificial tooth, and the extent of removal was dependent on the applied voltage setting and duration of the treatment.

**Fig. 4:**
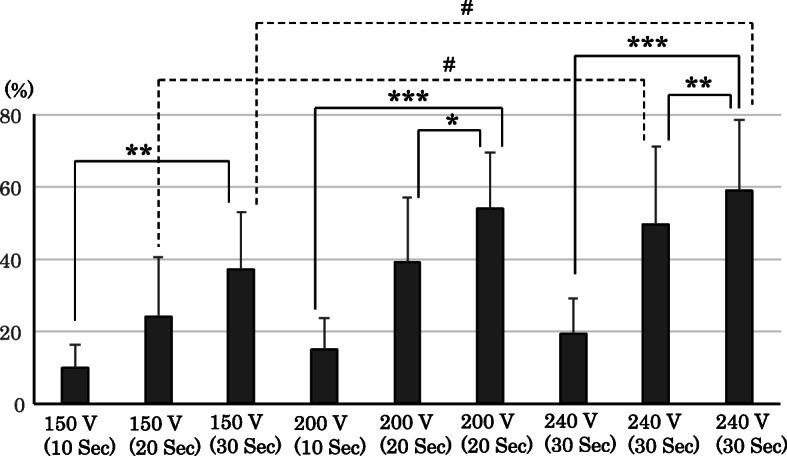
Comparison of the removal rates of artificial calculus among the different ADPJ settings. Difference by time *p < 0.05, **p < 0.01, ***p < 0.001 Difference by applied voltage #*p* < 0.05 ADPJ: actuator-driven pulsed water jet

Figure 4 presents the results of the comparisons in the mean artificial dental calculus removal rate, between the different combinations of the applied voltage settings and treatment durations. The removal rate was found to be highly dependent on both the applied voltage setting and treatment duration. In a comparison between treatment durations at a constant applied voltage setting of 200 V or 240 V, a 30 s treatment duration resulted in a significantly greater removal rate than those of 10 s and 20 s. At a constant applied voltage setting of 150 V, a significant difference was only observed between a treatment duration of 30 s versus 10 s. Among the comparisons between applied voltage settings with a constant treatment duration of 20 s, a significantly higher removal rate was observed for 240 V compared to 150 V. Similarly, at a constant treatment duration of 30 s, the removal rate was significantly greater for 240 V compared to 150 V (all *p* < 0.05).

### Surface of the artificial teeth and apatite pellets

**Fig. 5:**
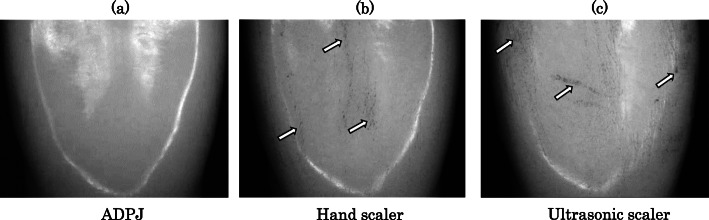
Microscopic images of the surface of the artificial tooth. **a** After removal the artificial calculus using the ADPJ. **b** After removal the artificial calculus using the hand scaler. **c** After removal the artificial calculus using the ultrasonic scaler. ADPJ: actuator-driven pulsed water jet

As shown in Fig. 5a, the Evans blue dye was not visible on the surface of the artificial tooth treated with ADPJ. In contrast, it was clearly retained on the surfaces of the artificial teeth treated with the hand (Fig. 5b) and ultrasonic scalers (Fig. 5c).

**Fig. 6:**
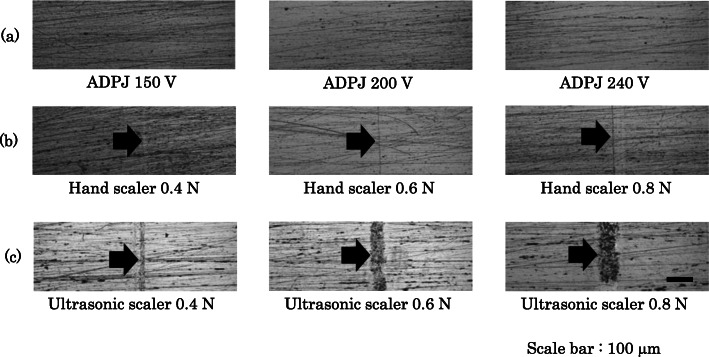
Laser microscopic images of the surface of the apatite pellets. **a** After treatment with the ADPJ. **b** After application of the hand scaler. **c** After application of the ultrasonic scaler ADPJ: actuator-driven pulsed water jet

Figure 6 shows that the laser microscopic images of the surfaces of apatite pellets after treatment with the ADPJ, hand scaler and ultrasonic scaler. A scratch mark was not observed on the surface of the apatite pellets after ADPJ treatment (Fig. 6a). In contrast, a vertical scratch mark was observed after the use of both the hand (Fig. 6b) and the ultrasonic scalers (Fig. 6c).

## Discussion

The aim of this study was to test the hypothesis that the ADPJ could remove dental calculus without damaging the underlying enamel surface. The use of artificial teeth and dental calculus facilitated the standardization of experimental variables between groups, as the level of existing calculus is widely variable on natural teeth. Indeed, Sawaguchi et.al [12]. reported that the removal force variation was smaller in the artificial than the natural dental calculus adhered to the artificial and human teeth, respectively.

In the first phase of this study, we developed a high-voltage ADPJ with a maximum setting of 240 V; this was modified from our previous device, which was designed to remove lesions on soft tissues (e.g., liver; 40–80 V, internal thoracic artery; 80–100 V) [9, 10]. We demonstrated that the effectiveness of dental calculus removal by the ADPJ at a constant jet pressure was dependent on the applied voltage (Fig. 3), and that a high voltage ADPJ would be required for periodontal treatment in a clinical setting. Previous studies have reported that the cutting ability and speed of a pulsed jet on harder materials is greater compared with the continuous jet operating at the same parameters (i.e., nozzle diameter, operating pressure, and output power) [13]. These findings may explain why a pulsed water jet can efficiently dissect tissues with a relatively smaller amount of water and energy. It is possible that accumulated water on the tissue surface affects the dissecting ability, and it is necessary to study this effect in the future. It has previously been shown that water released from the tip of the ADPJ can remove a tumorous lesion, while leaving the surrounding normal tissues unharmed [14]**.** The results of the present study suggest that it is possible for the pulsed water flow to penetrate and accumulate in between the artificial tooth surface and the attached artificial calculus, and effectively dislodge the latter.

In order to evaluate damage to the tooth surface, both artificial teeth and apatite pellets were used. The rationale for using apatite pellets was that scratch marks could be accurately evaluated, because the original apatite pellets were free of any scratch marks; this is not the case with extracted human and animal teeth, which are frequently used in in vitro investigations [11]. The present study demonstrated that the ADPJ was able to remove the artificial dental calculus without damaging the artificial teeth (Fig. 5); this result would also apply to natural tooth enamel, as the surface of an artificial tooth, with components of melamine resin, is much softer. In fact, apatite pellets which had the same hardness as enamel remained undamaged following the use of the ADPJ (Fig. 6). The lack of damage to the substrate surfaces may be attributed to the use of a water stream by the ADPJ system. In contrast, both the hand and ultrasonic scalers made scratch marks on the apatite pellets, reflecting the inevitable damage that occurs when scalers are used to remove dental calculus directly adhered to natural tooth surfaces in vivo. This is especially pertinent for ultrasonic scaler systems, in which the primary mechanism for calculus removal is the mechanical chipping action of the oscillating scaler probe when being in contact with the tooth surface [3]. While the use of an Er: YAG laser for calculus removal has been reported [6, 7], Aoki et al. observed enamel damage in the form of vaporization and roughness on scanning electron microscope imaging, after the use of an Er: YAG laser on beagle dog premolars [5].

The results of this study suggest that the ADPJ may be used safely for the removal of calculus. There are some limitations to this study. First, the effectiveness of the ADPJ in removing natural dental calculus is not clear, as we have used an artificial model in this study. Considering the different material properties between the artificial and natural dental calculus, further studies will be needed to evaluate ADPJ effectiveness on natural teeth. In addition, the use of high-voltage ADPJ for the removal of calculus adjacent to soft tissues (e.g., in the case of subgingival calculus is adhered to the tooth root surface) requires additional safety evaluations. Prior to clinical use, it is necessary to determine the effect of applying a high voltage to the soft tissues and tooth root surfaces at various nozzle positions, distances, and angles in animal experiments. The observation of root surface damage or soft tissue damage (i.e., bleeding or inflammation) would necessitate a readjustment of the applied voltage, frequency, and water volume. Furthermore, long-term clinical studies comparing the prognosis of treated teeth after the use of ADPJ and conventional scalers are required to substantiate the clinical effectiveness of the pulsed water jet system.

## Conclusions

We evaluated the effectiveness of the ADPJ in removing artificial dental calculus as well as the surface features of artificial teeth and apatite pellets. The results of this study demonstrated the ability of the ADPJ to remove artificial dental calculus without damage to the surface of artificial teeth and apatite pellets. Further studies on calculus adhered to natural teeth are required to substantiate the clinical effectiveness of the ADPJ, and its potential to be integrated into routine periodontal therapy.

## Data Availability

The datasets used and/or analyzed during the current study are available from the corresponding author on reasonable request.

## Abbreviations

ADPJ: Actuator-driven pulsed water jet
Er:YAG laser: Erbium-doped yttrium aluminum garnet laser
CP: Stainless steel connecting pipe
Di: Stainless steel diaphragm
PA: Piezo electric actuator
